# Integrative molecular network analysis identifies emergent enzalutamide resistance mechanisms in prostate cancer

**DOI:** 10.18632/oncotarget.22560

**Published:** 2017-11-20

**Authors:** Carly J. King, Josha Woodward, Jacob Schwartzman, Daniel J. Coleman, Robert Lisac, Nicholas J. Wang, Kathryn Van Hook, Lina Gao, Joshua Urrutia, Mark A. Dane, Laura M. Heiser, Joshi J. Alumkal

**Affiliations:** ^1^ Department of Biomedical Engineering, OHSU Center for Spatial Systems Biomedicine, Oregon Health and Science University, Portland, OR 97239, USA; ^2^ Knight Cancer Institute, Oregon Health and Science University, Portland, OR 97239, USA

**Keywords:** prostate cancer, castration resistant, drug resistance, enzalutamide

## Abstract

Recent work demonstrates that castration-resistant prostate cancer (CRPC) tumors harbor countless genomic aberrations that control many hallmarks of cancer. While some specific mutations in CRPC may be actionable, many others are not. We hypothesized that genomic aberrations in cancer may operate in concert to promote drug resistance and tumor progression, and that organization of these genomic aberrations into therapeutically targetable pathways may improve our ability to treat CRPC. To identify the molecular underpinnings of enzalutamide-resistant CRPC, we performed transcriptional and copy number profiling studies using paired enzalutamide-sensitive and resistant LNCaP prostate cancer cell lines. Gene networks associated with enzalutamide resistance were revealed by performing an integrative genomic analysis with the PAthway Representation and Analysis by Direct Reference on Graphical Models (PARADIGM) tool. Amongst the pathways enriched in the enzalutamide-resistant cells were those associated with MEK, EGFR, RAS, and NFKB. Functional validation studies of 64 genes identified 10 candidate genes whose suppression led to greater effects on cell viability in enzalutamide-resistant cells as compared to sensitive parental cells. Examination of a patient cohort demonstrated that several of our functionally-validated gene hits are deregulated in metastatic CRPC tumor samples, suggesting that they may be clinically relevant therapeutic targets for patients with enzalutamide-resistant CRPC. Altogether, our approach demonstrates the potential of integrative genomic analyses to clarify determinants of drug resistance and rational co-targeting strategies to overcome resistance.

## INTRODUCTION

Prostate cancer is the third leading cause of cancer-related death in the United States [1]. Nearly all of these deaths are due to castration-resistant prostate cancers (CRPC), the lethal form of the disease that has progressed despite androgen deprivation therapies that interfere with androgen levels or androgen receptor (AR) function [2]. Recent work demonstrates that CRPC cells develop resistance to androgen deprivation therapy by synthesizing their own androgens or making use of adrenal androgens to sustain AR function [3, 4, 5]. These discoveries led to the development of the novel anti-androgen drug enzalutamide, which competes with androgens for binding to AR and leads to suppression of CRPC tumor growth in pre-clinical models [6]. Recently, two phase III clinical trials demonstrated that enzalutamide treatment significantly improves overall survival in CRPC patients, and enzalutamide is now approved for the treatment of men with metastatic CRPC [7, 8]. However, nearly half of all patients do not respond to enzalutamide treatment, and resistance is universal [9].

Several emergent resistance mechanisms to enzalutamide have been identified. These include AR splice variants that lack the ligand-binding domain for androgens or enzalutamide as well as F876L mutations in the ligand-binding domain that may convert enzalutamide to an agonist [10-12]. The prevalence of F876L or other enzalutamide resistance-associated mutations has been reported to occur in approximately 20% of CRPC patients progressing on enzalutamide [13, 14]. Although these mutations are clinically-relevant examples of acquired drug resistance, these mechanisms are currently not targetable by therapeutic compounds. In addition to AR mutations, CRPC tumors harbor countless genomic aberrations that control many hallmarks of cancer [15-17], however most of these are not actionable drug targets [17]. Therefore, there is an urgent need to further clarify mechanisms of enzalutamide resistance in order to identify rational strategies to overcome enzalutamide resistance. Further, an enhanced understanding of the genetic basis of drug resistance and treatment failure may lead to better molecular stratification of patients for treatment, as well as to predict prognosis.

Our prior work demonstrated that the genomic aberrations in cancer operate in concert to promote drug resistance and tumor progression [18, 19]. We hypothesized that organizing genomic aberrations into biologically meaningful pathways may improve our ability to understand mechanisms of resistance to enzalutamide treatment. Here, we performed genomic studies using paired enzalutamide-sensitive and resistant LNCaP cell models. After transcriptional and copy number profiling, we performed integrative pathway analysis using PARADIGM to identify differentially regulated cellular networks [18, 19] and analyzed these large-scale networks to identify sub-networks associated with acquired resistance. Genes residing within significant sub-networks were nominated for functional validation studies with RNAi. We identified specific sub-networks that contribute to enzalutamide resistance. Importantly our approach identified nodes in these sub-networks that may be targeted therapeutically, demonstrating the translational significance of this approach.

## RESULTS

### Enzalutamide-resistant cells have distinct genomic profiles as compared to parental enzalutamide-sensitive cells

**Figure 1:**
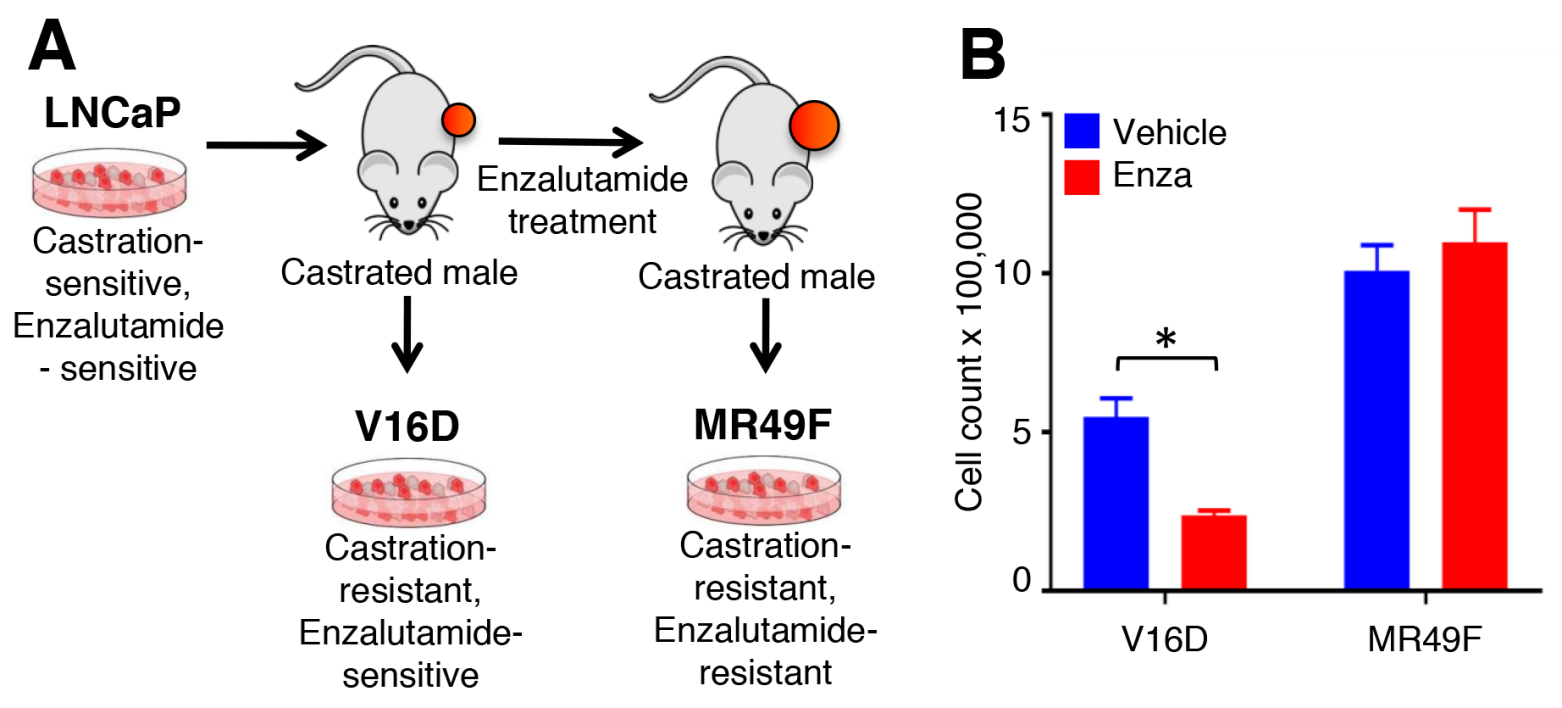
Cell line models of enzalutamide resistance **(A)**. Castration-sensitive, enzalutamide-sensitive human LNCaP cell line underwent serial murine xenograft transplantation in the setting of androgen deprivation therapy to produce a castration-resistant but enzalutamide-sensitive V16D cell line model. Continued xenograft transplantation in the presence of enzalutamide gave rise to the castration-resistant and enzalutamide-resistant MR49F cell model. **(B)**. V16D and MR49F cell counts after 5 days of 10 μM enzalutamide treatment confirm that the MR49F cell line is resistant to enzalutamide while the V16D cell line remains sensitive. Bars represent the mean of the Invitrogen Countess automated cell count. ^***^ denotes statistical significance (p < 0.05).

**Figure 2:**
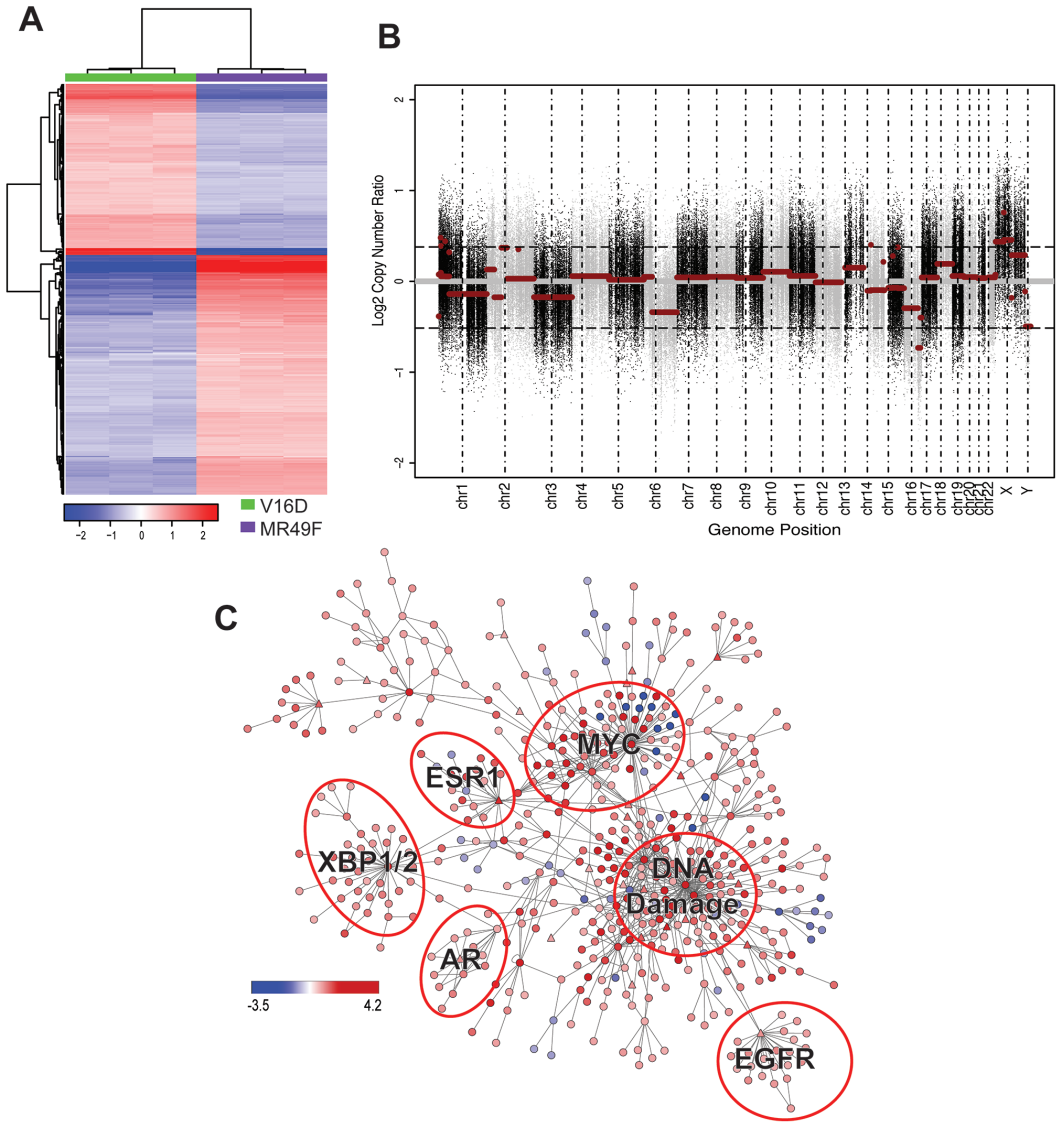
Molecular profiling of MR49F **(A)**. Heatmap showing the 586 differentially expressed genes with a fold-change > 2 between MR49F and V16D (FDR-adjusted q < 0.05). Color reflects median centered expression values. **(B)**. Copy number aberrations in cell line MR49F relative to V16D. Each dot represents the log2 ratio between MR49F and V16D cell lines for one probe region of the captured exome. Horizontal red lines represent the normalized log2 ratio after segmentation. **(C)**. Differentially regulated PARADIGM network rendered with Cytoscape. Node color represents PARADIGM-inferred IPL scores where red indicates up-regulated in and blue indicates down-regulated in MR49F as compared to V16D. Triangle-shaped nodes indicate druggable targets. Biologically meaningful deregulated subnetworks are indicated.

To understand the molecular basis of enzalutamide-resistance in CRPC, we utilized the V16D enzalutamide-sensitive CRPC cell line, as well as MR49F, its enzalutamide-resistant derivative. These cell lines were derived by long-term treatment of LNCaP-CRPC xenografts with enzalutamide for multiple generations to create the enzalutamide-resistant cell line MR49F (Figure 1A) [20]. To establish the baseline therapeutic response of these cell lines, we cultured V16D cells or MR49F cells in growth media supplemented with either vehicle or 10 μM enzalutamide and measured cell viability. As expected, we observed that enzalutamide treatment reduced viability of V16D cells but did not reduce viability of MR49F cells, demonstrating their resistant phenotype (Figure 1B). Further, enzalutamide treatment of V16D cells suppressed the expression of canonical AR target genes while there was no effect in MR49F cells, in keeping with the fact that MR49F cells harbor an AR F876L resistance-conferring mutation [19]. We performed RNA sequencing and exome sequencing to identify the gene expression differences and copy number changes between the V16D and MR49F cell lines. This analysis identified 586 significant differentially expressed genes between V16D and MR49F cells (*t*-test, q < 0.05, fold-change > 2, Figure 2A, Supplementary Table 1). Gene-set enrichment analysis demonstrated that the top deregulated pathways include those linked to MEK, EGFR, the RAS-associated kinase STK33, and TBK1 a serine-threonine protein kinase associated with NFKB signaling (Supplementary Table 2).

We reasoned that copy number changes specifically present in the resistant MR49F cell line as compared to the parental V16D cell line were candidate resistance-associated genomic changes [21]. Our analysis revealed focal copy number gains and losses on multiple chromosomes of MR49F as compared to V16D, including regions of significant amplification on chromosomes 1 and X, and a deletion on chromosome 16 (Figure 2B, Supplementary Tables 3 and 4). The focal gain on chromosome 1 contains just a few genes, including MTHFR, a gene implicated in folate metabolism. The amplification on chromosome X is comprised of 3 significant regions and includes AR.

### PARADIGM network analysis identifies pathways deregulated in enzalutamide resistant cells

We next sought to identify the pathways and gene networks that were differentially expressed in MR49F cells as compared to V16D cells. We used PARADIGM, a network modeling approach that integrates multiple data types to infer alterations in biological pathway activity that contribute to a specific phenotype or cell state [19]. PARADIGM inferred from the molecular profiling data an Integrated Pathway Level (IPL) score for each node in a reference pathway curated from Reactome, BioCarta, and NCI Nature pathway resources [37]. A key feature of PARADIGM is that the IPL for each node reflects both the input data (e.g., gene expression and copy number) as well as the scores of neighboring nodes. In our analysis, we used the transcriptional profiling and copy number data to infer a network associated with enzalutamide resistance. This PARADIGM network revealed that ER, EGFR, MYC, and DNA Damage pathways were differentially-regulated between the parental V16D CRPC cell line and the enzalutamide-resistant MR49F cell lines (Figure 2C).

We further analyzed this large network to identify biologically relevant deregulated pathways by filtering the PARADIGM results for nodes that showed a change in pathway activity greater or less than 2 standard deviations from the median IPL score. This approach nominated 607 nodes with 235 nodes representing individual genes. We reasoned that genes with the greatest differential-expression between V16D and MR49F would be most important for mediating resistance. After applying a filter to remove lowly expressed PARADIGM-nominated genes, we identified 64 genes for functional validation with RNA interference. We also included as biological controls several genes known to be important for prostate cancer cell viability: AR, MYC, and KIF11 (Supplementary Table 5).

### RNAi screen identifies critical pathway nodes that contribute to enzalutamide resistance

To identify genes that promote survival and contribute to enzalutamide resistance in MR49F cells, we used RNAi to suppress the 64 nominated genes. We also measured cell viability in V16D cells after suppression of these same genes, to allow us to identify genes whose suppression was specific to the enzalutamide-resistant MR49F cell line. Cell viability was quantified with MTS assays, and data were normalized to quantitate the change in viability after knock-down (Supplementary Table 6).

**Figure 3:**
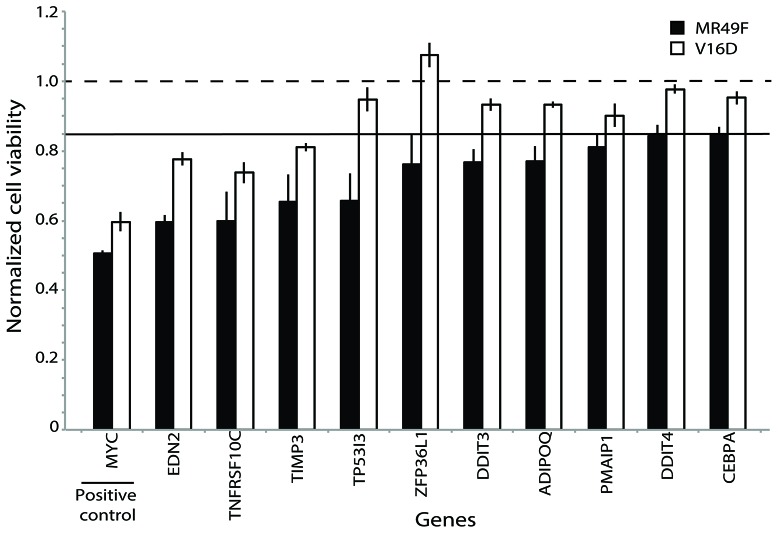
Functional validation of PARADIGM-nominated gene candidates identifies genes mediating enzalutamide resistance Bars represent viability in V16D or MR49F cell lines after RNAi knock-down with specific genes, where viability scores have been normalized against scramble control. Horizontal line at 0.85 represents the minimum viability effect required to indicate a functional hit. Error bars represent s.e.m.

**Figure 4:**
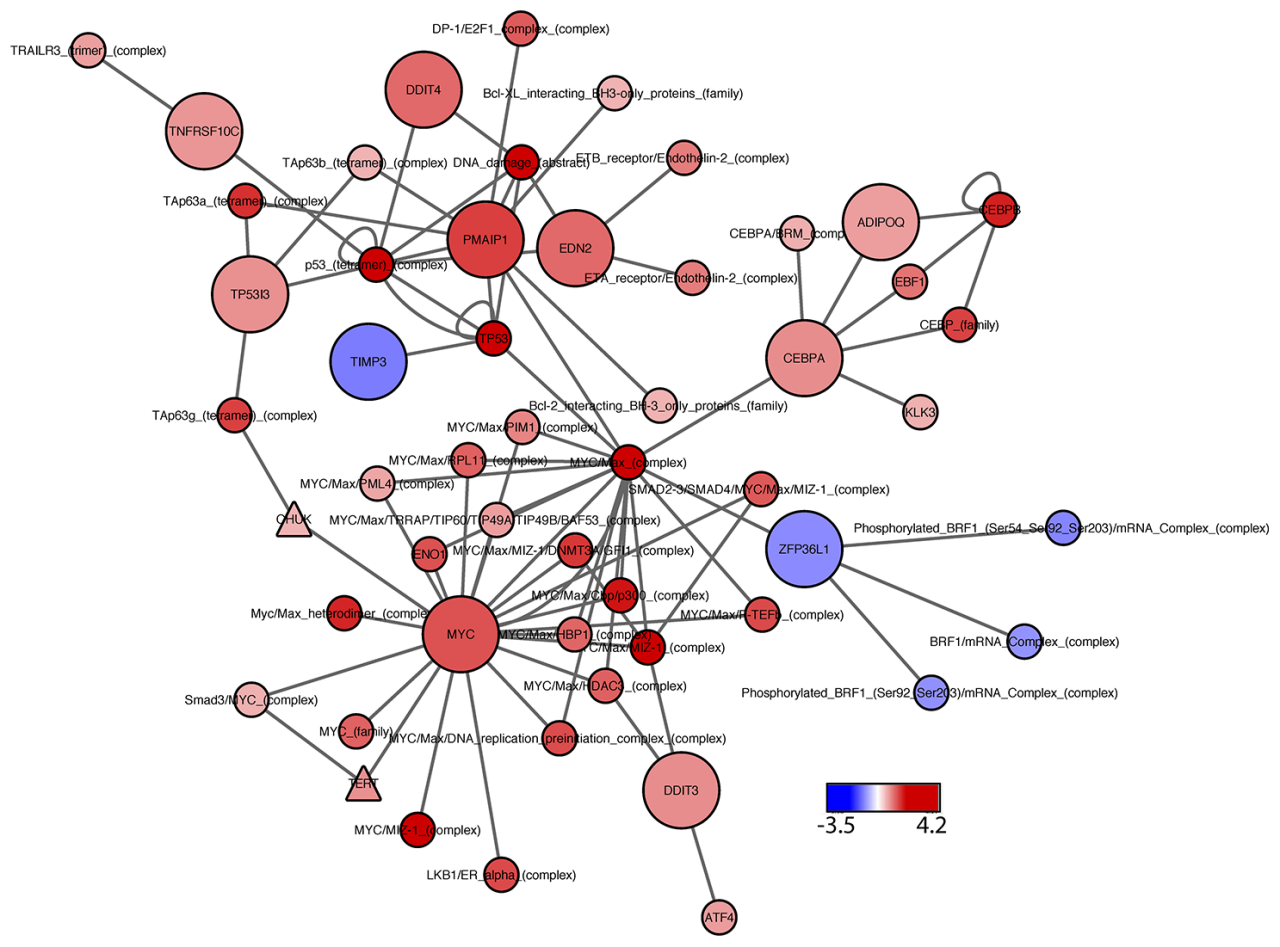
PARADIGM subnetwork for top-hit genes Network includes the statistically significant siRNA hits, positive control (MYC), and their first neighbors, as identified from the PARADIGM curated pathway information file. Node color represents PARADIGM network scores, where blue indicates down-regulated activity in MR49F as compared to V16D, and red indicates upregulated network activity. Node shape: circles represent PARADIGM network entities, with significant siRNA hits indicated by large circles; triangles indicate drug targets.

Next, we attempted to home in on the PARADIGM-nominated genes causally associated with enzalutamide resistance by filtering the RNAi output to identify genes that caused at least a 15% decrease in cell viability in the MR49F cell line, and also showed a greater decrease in cell viability in the MR49F cell line as compared to the V16D cell line. This approach identified 10 genes as functionally associated with enzalutamide resistance: EDN2, TP5313, TNFRSF10C, TIMP3, ZPF36L1, DDIT3, ADIPOQ, PMAIP1, DDIT4, and CEBPA. The control gene MYC was also identified as having preferential cell viability effect in MR49F as compared to V16D (Figure 3, Supplementary Table 7). AR knock-down had a modest effect on MR49F, though it was below the threshold we set to identify hits. We assessed the knockdown hits in the context of the computed PARADIGM network and found that many of them reside in inter-connected networks inferred to be upregulated in the MR49F enzalutamide resistant cell line as compared to the enzalutamide sensitive cell line (Figure 4). This indicates that these gene alterations coordinately contribute to enzalutamide resistance and moreover suggests that targeting key nodes in enzalutamide-resistant tumors may block this network.

**Figure 5:**
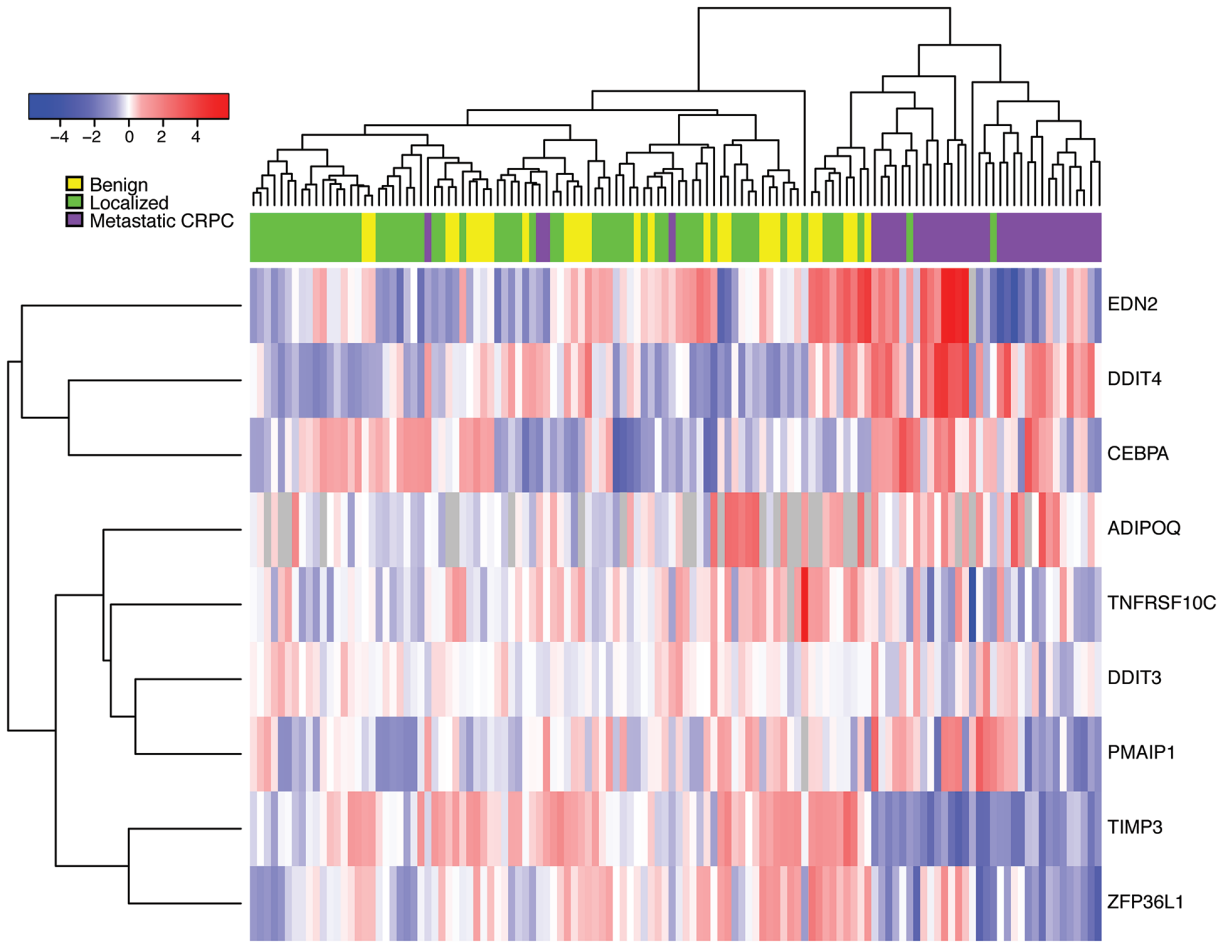
Expression of nominated siRNA hits in CRPC tumors, primary tumors, and benign prostate samples Heatmap represents gene expression for CRPC primary tumors from Grasso, *et al.* after median-centering. Metastatic CRPC samples (purple) cluster together after unsupervised hierarchical clustering, indicating that the siRNA hits implicated in enzalutamide resistance are coordinately deregulated in these samples.

We next sought to determine the clinical significance of hits identified from our pre-clinical studies. Publically available datasets of enzalutamide-sensitive and resistant cells were not available, so instead, we assessed the expression of our 10 “top hit” genes in a cohort of human samples comprised of metastatic CRPC and localized prostate cancer samples [12]. Importantly, several of the top-hit genes were more highly expressed in the metastatic CRPC samples as compared to the localized prostate cancers (Figure 5). These genes include: ADIPOQ, CEBPA, and DDIT4. We also observed concordant down-regulation of expression of TIMP3 and ZFP36L1 in both the enzalutamide-resistant MR49F cell line and metastatic CRPC prostate cancers. Taken together, these findings demonstrate the relevance of our *in vitro* studies and suggest that these pathway nodes may already be selected for in the transition from hormone-responsive prostate cancer to CRPC tumors.

## DISCUSSION

In recent years, the knowledge that androgens persist in CRPC cells and are sufficient to activate AR transcriptional activity has led to the development of new therapies to treat patients with CRPC. The second-generation AR antagonist enzalutamide is one such example, and two phase III clinical trials demonstrate that enzalutamide improves survival for patients with metastatic CRPC [7, 8]. However, even patients who initially respond to enzalutamide ultimately undergo disease progression, demonstrating the urgent need to identify mechanisms of acquired resistance.

Several enzalutamide resistance mechanisms have been described previously, including both AR-dependent and AR-independent alterations. Examples of the former include: AR splice variants that lack the ligand-binding domain [22], gain of function AR mutations [13, 14], AR gene amplification [13, 14], the glucocorticoid receptor maintaining AR signaling [23], and reciprocal feedback between the AR and the PI3K pathways [24]. Examples of AR-independent mechanisms are much less well described, although the clinical frequency of neuroendocrine prostate cancer appears to be on the rise, perhaps due to the selective pressure of treatment with drugs such as enzalutamide that block AR function [25, 26]. MYC has also been implicated in CRPC, as demonstrated by both genomic alterations [27] as well as demonstration that androgen treatment leads to downregulation of MYC and concomitant upregulation of both IGF1 and EGFR [28]. It is clear that a multiplicity of gene alterations is common in CRPC tumors, including in those resistant to enzalutamide. A better understanding of the importance of specific genomic alterations and how they cooperate could aid with patient stratification and treatment selection.

In this report, we used the PARADIGM algorithm to identify emergent enzalutamide resistance mechanisms that could be targeted to improve therapeutic response. The PARADIGM algorithm is based on the premise that pathway level analysis provides better sensitivity and specificity in identifying relevant oncological alterations in comparison to approaches that consider genes as independent actors. One distinct advantage of PARADIGM is the ability to simultaneously integrate multiple genomic and epigenomic data types, providing an analytical framework for integrating molecular changes that occur across different omic levels -- from DNA to RNA to protein. Furthermore, PARADIGM superimposes biological data with curated molecular pathways from the literature. This enables inference of genetic or epigenetic alterations that are likely to be functional contributors to a specific cell state or phenotype and to identify critical interdependencies that occur at the pathway level. We leveraged these ideas to understand the coordinate copy number and transcriptional changes that occur with enzalutamide resistance.

We focused on pathway analyses because this approach can identify genes implicated in specific networks or processes, however, this approach has some general limitations. Most critically, pathway-based analyses rely on a curated pathway definition and, as such, may ignore deregulated genes that are not well curated [29]. Furthermore, there are many different algorithms available for pathway analysis, each with their own strengths and weaknesses: ARACNe and MARINA to discover and interrogate transcription factor networks [30-32], HotNet which uses heat diffusion to model the effects of gene alterations [33], and signaling pathway impact analysis (SPIA) to identify highly impactful nodes in a network [34]. Nonetheless, our pathway-based approach identified important genes linked to enzalutamide resistance. While computational approaches such as these can aid in understanding biological mechanisms, it is important to note that they are limiting in that they can only identify associations with phenotype. Specifically, functional experimental validation is critically necessary for identifying causal relationships between pathway activity and cellular function.

Functional validation of our top-hit genes with RNAi identified several genes that conferred survival to an enzalutamide resistant cell model, suggesting that genes—or the networks in which they reside—may be important contributors to enzalutamide resistance. These functional validation studies identified PARADIGM-nominated genes that are more critical for conferring cell viability in enzalutamide-resistant MR49F cells as compared to parental V16D cells. It is worth noting, however, that several of these genes reduced viability in the enzalutamide-sensitive V16D cell line as well, suggesting that they may be important for mediating both enzalutamide resistance and viability in castration-resistant prostate cancer cells.

Examination of CRPC and hormone-naïve prostate cancer datasets demonstrated that several of our functionally-validated hits are deregulated in metastatic CRPC tumor samples, suggesting translational potential of our studies. For example, CEBPA is a transcription factor involved in cell cycle regulation and tissue differentiation. A recent study found that CEBPA promotes castration-resistance and AR signaling in prostate cancer through a direct protein interaction with AR; this suggests that co-targeting CEBPA and AR may be one approach to block AR in either castration-resistant or enzalutamide-resistant tumors [35]. Consistent with our functional studies, PMAIP1 (NOXA), which promotes activation of caspases and apoptosis, is associated with CRPC, indicating that PMAIP1 may be another promising target to overcome enzalutamide resistance [36]. Interestingly, we observed variable expression of many of our top-hit genes across a cohort of primary tumor samples, which suggests molecularly distinct subsets of CRPC. It is worth noting that the cohort of tumor samples we assessed are not annotated with senstivitity or resistance to enzalutamide and it is likely that both types of patients exist in this sample set, providing a basis for the heterogeneous expression of some of these genes. For example, TP53I3 is regulated by the well-known cancer associated transcription factor TP53, and its response in our RNAi screen could indicate that the DNA damage pathway may be an effective therapeutic target in enzalutamide-resistant prostate cancer, for instance with PARP inhibitors. A prior network analysis nominated ZFP36L1 as a critical gene in metastatic prostate cancer, and our functional studies validated that prediction [37]. We observed that enzalutamide-resistance was associated with down-regulation in expression of TIMP3 and ZFP36L1, and that this pattern was also shared in CRPC tumors. This finding is consistent with the idea of CYCLOPS genes that have undergone copy number loss and are therefore vulnerable to knock-down [38]. Altogether, our studies reveal an interconnected gene network associated with enzalutamide resistance in CRPC.

Our PARADIGM and gene set enrichment analyses revealed that enzalutamide resistance is associated with MEK, EGFR, ESR1, the RAS-associated kinase STK33, and TBK1 a serine-threonine protein kinase associated with NFKB signaling pathways. Several of these pathways are targetable with small molecule inhibitors, indicating that they may be directly therapeutically relevant. Consistent with our observations, a report by Toren et al [20] demonstrated that MR49F cells are more sensitive to the MEK inhibitor PD0325901 than are V16D cells. Several previous reports indicate expression of oncogene-specific tyrosine kinase signatures--including EGFR--in CRPC [39]. Further, expression of AR has been shown to be negatively regulated by EGFR and ERBB2 in CRPC cells [40]. Consistent with this, the MR49F cell line is sensitive to the EGFR/ERBB2 inhibitor lapatinib, both *in vitro* and *in vivo* [41]. These studies highlight the ability of our approach to identify therapeutically-relevant networks that may be targeted to improve outcomes for patients with enzalutamide-resistant prostate cancer.

In our viability studies, we tested a single time point of 5 days post-treatment. While this is a biologically meaningful time in which to observe changes associated with therapy or RNAi knock-down, it does not capture information about the dynamic nature of therapeutic response. Future studies that focus on identification of early and late responses will lead to greater insights about the nature of therapeutic resistance and will provide additional insights into how to improve therapeutic responses [42]. An additional limitation to our RNAi studies is that they focus on a singular phenotypic change of cell viability. While this is one of the most clinically relevant changes induced by therapeutic treatment, it does not capture phenotypic changes that may affect other cancer hallmarks, such as differentiation state, DNA damage response, or migration [16]. Indeed, other studies that leverage high-content imaging have revealed other phenotypic changes in cells following therapeutic treatment [43].

The PARADIGM computational algorithm facilitates nomination of genes associated with the emergence of drug resistance―including mechanisms of enzalutamide resistance in CRPC. The integrative nature of this strategy goes has clear advantages over traditional means of comparative analyses, such as differential expression analysis, by providing biological context in which the gene alterations occur. In support of this are our functional data demonstrating the connectivity of many of our PARADIGM-nominated hits in specific gene networks. Improving patient stratification and understanding emergent resistance mechanisms is essential to improving clinical outcomes. In the future, we plan to examine the relevance of genes identified herein in tumor samples from CRPC patients undergoing enzalutamide treatment through a prospective clinical trial (NCT02099864). Once that trial is complete, PARADIGM analysis of pre- and post-treatment samples like the approach used here may enable us to confirm our findings and prioritize targets most relevant to overcome clinical enzalutamide resistance.

## MATERIALS AND METHODS

### Cell culture

Paired enzalutamide-sensitive and enzalutamide-resistant LNCaP derivative cell lines were generously provided by Martin Gleave from the University of British Columbia [44]. The enzalutamide-sensitive LNCaP-derived parental V16D cell line was maintained in RPMI 1640 (Gibco #11875-093) supplemented with 10% fetal bovine serum (FBS) (Gibco #16000-044). The enzalutamide-resistant MR49F cell line was constitutively maintained in RPMI 1640 supplemented with 10% FBS and 10 μM enzalutamide (MedchemExpress #HY-70002). Cells were passaged by treatment with trypsin with EDTA and phenol red (ThermoFisher Scientific #R001100). Each cell line was processed and analyzed for transcriptional and copy number profiling in biological triplicate.

### RNA-sequencing and analysis

MR49F and V16D cells were seeded in RPMI and 10% FBS in biological triplicate in 6-well plates at 300,000 cells per well. After 96 hours, or 50% confluence, cells were treated with 10 μM Enzalutamide or 0.1% DMSO and harvested 24 hours post treatment. Total cellular RNA was extracted with Trizol/CHCl3 and re-suspended in 30uL RNAse-free water. Cell line RNA libraries were prepared for sequencing with the Agilent SureSelect RNAseq protocol as per the manufacturer’s instructions. Triplicate samples were sequenced on an Illumina HiSeq as single-end 50 bp reads. Samples were aligned to the human reference (hg19) using Tophat (v2.0.9) and transcripts were assembled and quantified by Cufflinks (v2.1.1). To identify significant differentially expressed genes between V16D and MR49F samples, we performed a *t*-test, followed by multiple comparisons correction with Benjamini-Hochberg false discovery rate (q-value < 0.05 deemed significant). Additionally, to ensure identification of a biologically meaningful set of differentially expressed genes, we required a minimum fold-change of 2 between V16D and MR49F cell lines.

### Whole exome sequencing and analysis

Cellular DNA from V16D and MR49F cell lines was extracted using the Agilent SureSelect XT2 protocol. Exonic regions were isolated by hybrid capture for Agilent’s 71Mb + UTR V5 library. Samples were sequenced on an Illumina HiSeq as paired-end 100 bp reads at an average depth of 30X. Data were aligned to the human genome (hg19) using bwa (v0.7.3a), duplicates were sorted and removed with samtools 0.1.19 and Picard tools 1.51 and local realignment and quality score recalibration was done with GATK version 2.1-13. Copy number analysis was performed using DNACopy (v1.44.0) in R (v3.2.3) [45]. The parental V16D cell line was used as the comparator in the copy number analysis.

### PARADIGM analysis

We used RNAseq derived expression and gene-level copy number data as input to the PARADIGM algorithm. To generate a single expression value representing a differential between our samples of interest, the expression data was first log2 transformed and subtracted from the corresponding parental cell line’s expression levelfor each gene. Copy number data was preprocessed as log2 copy number ratios on a per-gene basis, comparing the derived to parental cell line. We analyzed these data with the PARADIGM algorithm webtool available through Five3 Genomics (w). We used the pid_120912 curated network file and visualized the resultant networks with Cytoscape v.3.1.1 [46].

### Nominating PARADIGM siRNA candidates

We considered PARADIGM-nominated genes with integrated pathway level (IPL) scores greater or less than 2 standard deviations from the median to be most biologically relevant. In an effort to prioritize the 235 nodes that passed the IPL cutoff criteria, we reasoned that genes with low or invariant expression could be excluded as RNAi candidates. We eliminated these genes by implementing a coefficient of variation filter that removed genes with CVs of less than 10 and greater than 200. We also required non-zero gene-level expression in at least two samples. This yielded 64 gene candidates for functional validation with RNAi. In addition to the 64 PARADIGM-nominated gene candidates, we included in our RNAi screen several genes as biological controls: scramble as negative control, KIF11 as positive control for viability, as well as MYC and AR because they have been implicated in CRPC and enzalutamide-resistance.

### RNAi screen

Gene candidates nominated from our PARADIGM analysis were validated with RNAi. Briefly, for both the V16D or MR49F cell lines, 5000 cells per well were seeded in a 96-well microtiter plate pre-coated with specific oligo RNA sequences in RPMI + 10%FBS + 10 μM enzalutamide. After 24 hours, RNAi transfection was initiated by addition of lipofectamine (ThermoScientific #T2002-01) to each well containing a unique smart pool of 50 μM siRNA probes (Dharmcon). After 6 hours, the volume of each well was doubled by addition of enzalutamide-containing cell media to cease the transfection by dilution. Next, each microtiter plate was allowed to incubate at 37 C without media change for five days to minimize cell loss. Cell viability was assessed by colorimetric MTS assay (Promega CellTiter 96 #G3581) per manufacturer recommended protocol by spectrophotometric absorbance at 490 nm. RNAi knockdown was conducted in biological triplicate per gene per cell line.

The MTS values were normalized as follows: for each plate, we created a bivariate loess model of the spatial variations using the formula MTS∼Row+Column with a span of 1 for plates where all wells were occupied by siRNAs and 0.2 for partially occupied plates. We divided each raw MTS value by its predicted loess value to generate a normalized estimate of cell viability that removed any spatial artifact. We then divided these normalized values by the normalized value of the non-targeted negative control from the same plate. The plate median was used in lieu of the negative control in the one partial plate that did not have values for the non-targeted siRNA.

We used the following approach to identify siRNA hits of interest. First, we identified genes that showed a significantly different knock-down effect in the two cell lines by performing a two-sided Wilcoxon rank sum test followed by Benjamini & Hochberg adjustment for multiple comparisons. Genes with q-values < 0.2 were further filtered to identify those with at least 15% cell kill in the MR49F cell line, as well as significantly greater cell kill in MR49F as compared to V16D.

## SUPPLEMENTARY MATERIALS TABLES

















## Abbreviations

CRPC: castration-resistant prostate cancer
AR: androgen receptor
IPL: integrated pathway level
PARADIGM: Pathway Representation and Analysis by Direct Reference on Graphical Models
