# Supplementary material for: Integrative molecular network analysis identifies emergent enzalutamide resistance mechanisms in prostate cancer
Source: Oncotarget. 2017 Nov 20;8(67):111084–95. doi: 10.18632/oncotarget.22560 (PMC5762307; doi:10.18632/oncotarget.22560)
Supplement: Supplementary file 1 [file oncotarget-08-111084-s001.pdf]

Integrative molecular network analysis identifies emergent enzalutamide resistance mechanisms in prostate cancer

SUPPLEMENTARY MATERIALS

Supplementary Table 1: Log2-transformed median-centered gene-level RNAseq expression data for two prostate cancer cell lines treated with MDV3100. Each cell line was tested in triplicate.

See Supplementary File 1

Supplementary Table 2: Gene Set Enrichment Analysis results for the 586 differentially expressed genes between enzalutamide-treated MR49F cells compared to enzalutamide-treated V16D cells.

See Supplementary File 2

Supplementary Table 3: MR49F copy number values in. seg format. Gene-level values are derived from the DNACopy algorithm with V16D as the comparator sample.

See Supplementary File 3

Supplementary Table 4: Genes contained within the copy number segments identified as significantly different between V16D and MR49F.

See Supplementary File 4

Supplementary Table 5: Molecular features for V16D and MR49F cell lines. A merged table comprised of copy number values, log2 expression values, PARADIGM scores, and Ensembl Gene IDs for genes that passed the initial variation filter. This table was used to prioritize genes for siRNA knockdown.

See Supplementary File 5

Supplementary Table 6: Raw and normalized siRNA scores for all genes tested. In total, 64 genes were tested for functional association with enzalutamide resistance. Additionally, 16 genes were included in the screen as part of another study; they are anonymized here. We have included all genes to allow reproducibility of loess normalization procedure described in methods.

See Supplementary File 6

Supplementary Table 7: Statistics used to identify siRNA knockdown hits. Hits were identified as having normalized MTS values < 0.85, and significantly greater knock-down effect in MR49F as compared to V16D.

See Supplementary File 7
